# Electroantennogram Responses of the Tea Slug Moth, *Iragoides fasciata* to Some Plant Volatiles Associated with Tea, *Camellia sinensis*

**DOI:** 10.1673/031.012.7501

**Published:** 2012-07-04

**Authors:** An-Ping Huang, Xiao-Cun Bao, Ben-Ying Liu, Yuan-Jiang Wang, Ling-Yun Zhou, J ing Ning, Bao-Yu Han

**Affiliations:** ^1^ Longping Branch of Graduate School, Central South University, Changsha, 410125, China; ^2^ Zhejiang Provincial Key Laboratory of Biometrology and Inspection & Quarantine of China Jiliang University, Hangzhou, 310018, China.; ^3^ Hunan Tea Research Institute, Hunan Academy of Agricultural Sciences, Changsha, 410125, China; ^4^ Tea Research Institute of Yunnan Academy of Agricultural Science, Menghai, 666201, China

**Keywords:** green leaf volatiles

## Abstract

Electroantennogram responses to a wide range of plant volatile compounds that have been identified in tea plants *Camellia sinensis* L. (Ericales: Theaceae) were recorded from males and females of the tea slug moth, *Iragoides fasciata* Moore (Lepidoptera: Limacodidae). The responses to 26 compounds, belonging to several chemical classes, and two mixtures were evaluated. The results showed significantly different electroantennogram responses to the different chemicals, as well as significantly different responses according to gender. The green leaf volatile components elicited significantly greater responses in males. In general, the antennae of males were more sensitive, and responded more strongly, to most of the compounds. Responses to sesquiterpenoids were lower in both males and females. Dose-dependent response studies indicated differences in response between genders and concentrations, suggesting the existence of sexual dimorphism. Compounds belonging to the green leaf volatiles class appeared to be important clues in host-plant selection by this oligophagous species.

## Introduction

The tea slug moth, *Iragoides fasciata* Moore (Lepidoptera: Limacodidae), which can break out under certain conditions, is one of the most destructive insect pests of tea (*Camellia sinensis* L. (Ericales: Theaceae)) in China. Its larvae generally feed on ripe leaves, but they can eat all the leaves of a tea plant when they outbreak, which leads to a loss of yields and profits for farmers. However, the larvae do not only eat act as a pest by eating leaves—they also sting people. Numerous data on *I. fasciata* biology, ecology, and control methods have been accumulated (Huang et al. 2009). Currently, chemical control is still the main strategy in integrated pest management (IPM). In view of the negative impact of chemical insecticides on health and the environment, a control method that is compatible with the environment should be added to the IPM system.

Host-plant selection by herbivores has been a major concern among researchers over the past decades. A number of valuable reviews have been reported on plant cues, and their effect on the behavior of herbivores (Denno et al. 1983; Visser 1986; Murlis et al. 1992; Rosenthal et al. 1992; Bernays et al. 1994; Howard 1995; Hay 1996; Dicke 2000; Bruce et al. 2005; Schoonhoven et al. 2005; Lastra et al. 2006; Powell et al. 2006; Finche et al. 2008). It is crucial for the successful development of its population for an herbivorous insect to find host-plants. Herbivorous insects have been shown to locate hosts by using a variety of host or habitat derived clues. Optical and odorous characteristics of plants are two important stimuli that could be used as directional cues (Schoonhoven et al. 2005). Olfactory cues are generally considered to be the most important clue for many phytophagous insects (Guerin et al. 1980; Fagoonee et al. 1983; Light et al. 1988; Cossé et al. 1995). Host-plant volatile compounds are a complex of hundreds of components. Olfactory cues may be used in IPM programs to control a pest species. A successfully established trapping technique may be used to monitor the presence, dispersion, and resistance to pesticides of an insect population, as well as to predict an upcoming infestation, or directly control a population (Karg et al. 1999). However, trapping technique could show promise for the suppression of pest population only after the development of attractive lures (Bengtsson et al. 2009).

In this paper, the olfactory sensitivity of males and females of *I. fasciata* to 26 plant volatile compounds and two mixtures was determined by a standard electroantennogram (EAG) method. The EAG is a bioassay widely used in experimental entomology. It can record the responses of many receptor neurons in the organ to the presentation of a stimulus, and thus allow for drawing sensitivity profiles for a series of chemicals, and differences in sensitivity between sexes (Thiéry et al. 1998). Plant volatiles emitted by their respective host-plants have shown to be of primary importance in host-plant selection in many phytophagous insects (Hern et al. 2002; Angioy et al. 2003; Piñero et al. 2007). GLVs, aromatic compounds including monoterpenes and sesquiterpenes, are olfactory clues in the process of host location in many insects (Ramachandran et al. 1990; Piñero et al. 2007; de Groot et al. 1990). Numerous studies have also shown that GLVs serve as modifiers of olfactory responses to sex pheromones (Dickens 1984; Dickens 1989; Dickens et al. 1993; Light et al. 1993; Groot et al. 2001; Ochieng et al. 2002, Tooker et al. 2002; Deng et al. 2004; Yang et al. 2004; Ginzel et al. 2005; Wang et al. 2008; Schmidt-Buesser et al. 2009), and have thus also been referred to as sexual kairomones (Ruther et al. 2002). Even though over 500 volatile components have been identified from the tea plant (Rawat et al. 2008), little is known about the role of these volatile compounds in the host-plant selection behavior of *I. fasciata*. The selection of the tested compounds was based on their distribution in the tea plant, Chemically related compounds and isomeric forms were included. Single compounds have been shown to be attractive to the adult moths in some species, but the evidence suggests that a complex of volatiles, rather than one single compound, is essential for olfactory orientation of herbivorous insect towards its host-plant (Piñero et al. 2007), Within Lepidoptera, mixtures of host-plant derived compounds are required to elicit appropriate levels of response in adult moths (Piñero et al. 2007). Based on this consideration, two mixtures mainly composed of GLVs and monoterpenes, or GLVs alone, were selected. The main aim of this investigation was to discover a short list of best-detected compounds that may either be attractants or repellents.

## Materials and Methods

### Insects

Fifth instar *I. fasciata* larvae were caught in a *C. sinensis* garden near the Tea Research Institute, Chinese Academy of Agricultural Sciences, and reared on tea leaves until they turned into cocoons. The cocoons were maintained in a cultivation cabinet under a 16:8 L:D cycle at 60% ±10% RH, 26 ±1 °C during the photophase and 22 ±1 °C during the scotophase. Moths were provided with a 10% sucrose solution. Moths of both genders (1–2 days old) were used for EAG recording experiments.

### Chemicals

Twenty-six plant volatile compounds (see Table 1) belonging to four classes, and two mixtures of them, were selected for EAG recordings. All the selected compounds have been identified in the tea plant (Ning et al. 1999; Wan 2007). Mixture 1 consisted of linalool, (E) -2-hexenal, and (Z) -3-hexen-É ol. Mixture 2 consisted of (E) -2-hexenal, (Z) -3-hexen-l-ol, 2-penten-l-ol, (E) -2-pentenal, (Z)-3-hexenol acetate, hexanol, and 1-penten3-ol. The mixtures were composed of all odor components listed above respectively at an equal ratio at concentrations of 10^-2^, 10^-4^ and 10^-6^ g/mL.

**Table 1.**
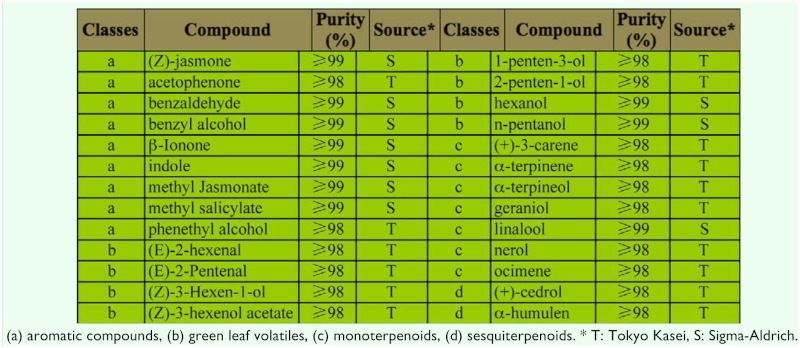
Diverse volatile compounds used for EAGs recordings in this study.

#### EAG Recordings

Antennal responses of adults of both genders to the 26 plant volatile compounds and two mixtures were investigated by a standard EAG method. The antennae of the adults (1–2 days old) were excised at the base by using micro scissors or a fine dissecting knife. A few segments of the antenna were also clipped off from the tip, in order to keep better contact. Then, the ends of the isolated antenna were connected to two stainless steel recording electrodes with Spectra 360 electrode gel (Spectra 360, www.parkerlabs.com/spectra360.html), which was applied to the metal electrodes surface in the field of view of a medium power stereomicroscope.

For EAG measurement, serial dilutions 10^-2^, 10^-4^, and 10^-6^ g/ml) of each test compound were made with paraffin oil (kerosene). The olfactory stimuli were prepared by applying 20 pi of chemical solution to a filter paper strip (6 × 0.5 cm). The solvent was allowed to evaporate for 30 seconds before the paper strip was inserted into a glass Pasteur pipette (15 cm long, 12 mm diameter). The small end of the pipette was inserted into the hole (3 mm diameter, 130 mm upstream from the outlet) in the mixing tube (12 mm diameter, 160 mm long), through which a continuous, charcoal filtered, and moistened airflow (600 mL/s) was blown onto the prepared antenna. Using a stimulus controller (model CS-55, Syntech Ltd. www.syntech.nl), a 0.1 second puff of charcoal filtered air (600 mL/s) was injected into the mixing tube through the Pasteur pipette, carrying the volatiles to the prepared antenna for stimulation.

EAG recording began 5 minutes after the antenna was prepared. Three controls were used: (1) a pipette containing filter paper, (2) a pipette containing 20µl paraffin oil only on filter paper, and (3) a pipette containing 20°l 10^-6^ g/mL linalool in paraffin oil on filter paper. The standard, primary experiment indicated that it could elicit an obvious antennal response. Presentation of the standard throughout the recording session permitted normalization of antennal responses. The EAG test procedures were similar to those described by Livy Williams (Williams et al. 2008). The following test protocol was arranged for each recording trial. The controls were measured in the following sequence: 1, 2, 3. Afterwards, three sample dilutions in a geometric sequence (10^-6^, 10^-4^, and 10^-2^ g/mL) were tested. Delivery of controls (3, 2) was made after each threeconcentration series of a sample dilution. After the final sample dilution for each recording, controls were presented in the following order: 3, 2, 1. At least 60 seconds were allowed between two stimuli in order to provide time for recovery of antennal responsiveness. Each antenna was tested with four or five series of sample dilutions. Each sample dilution was tested on 5 individuals of each sex. The EAG response was amplified, recorded, analyzed, and stored by the EAG apparatus (Syntech), which was linked to a desktop computer (with IDAC-2 data acquisition interface board). The maximum amplitude of depolarization elicited by a volatile stimulus using software from Syntech (EagPro Version 2. 0, Syntech) was defined as absolute EAG responses. All tests were conducted during the scotophase.

#### Statistical analysis

To compensate for artifacts, EAG values were standardized by expressing the corrected mean EAG values as a percentage of the standard stimulus. Data were analyzed by using the statistical software SPSS 10.0 for Windows.

Moth responses in the EAG experiment were compared by analysis of variance (ANOVA). Differences between male and female insects were analyzed by paired t-tests (p ≤ 0.05) at each dose of the tested compound.

### Results

#### Differentiation of EAG responses within different compounds

**Figure 1.**
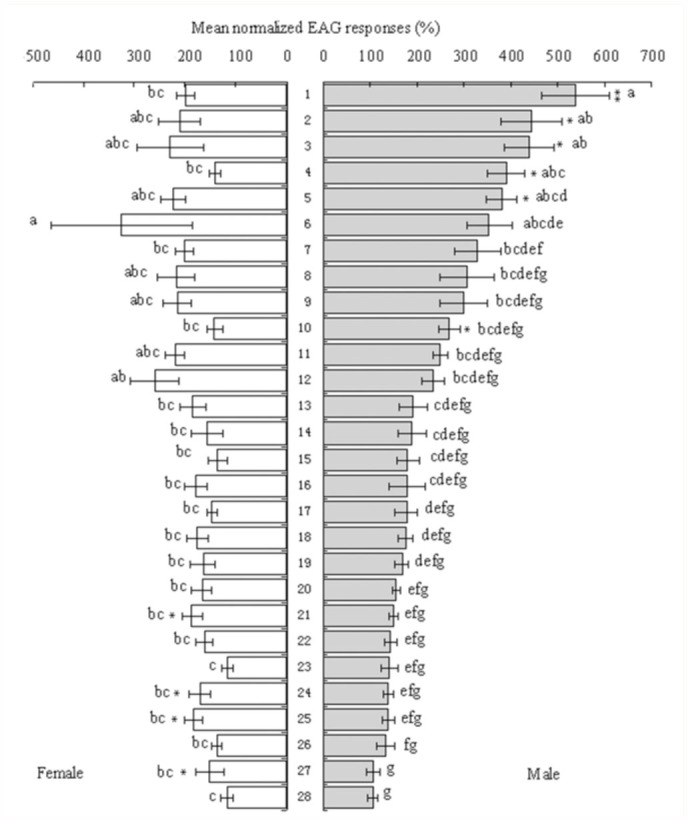
Mean normalized EAG responses of *lragoides fasciata* to plant volatiles. Responses of males (right) are ranked according to decreasing responses and females (left) in the same order as that obtained with males. EAG responses are expressed relative to the standard linalool (mean±.E., n = 15). Data are analysed by two-way ANOVA and the Duncan method of contrast. Bars marked with different letters indicate significant differences among plant volatiles (P<0.05). An asterisk indicates chemicals which elicited different relative EAG responses between males and females (^**^: p ^<^: 0.01, ^*^: p < 0.05). High quality figures are available online.

The antennae of both genders were tested with the 26 plant volatile compounds and two mixtures. Most tested compounds elicited a strong response (Figure 1). In general, the antennae of males were more sensitive, and had stronger responses to most of the compounds (F = 14.760, 1 df, P < 0.01). At the low concentration of 10^-6^ g/mL, the mean response of females to stimulation by linalool (standard) was 0.145 ± 0.075 mv, while it was 0.263 ± 0.082 mv in males (F = 0.327, 1 df, p > 0.05). The normalized response to the other chemicals varied in females, from 117.506 ± 6.738 (indole) to 325.701 ±142.672 (n-pentanol). In males, the mean relative values were much greater, and varied from 105.423 ± 6.347 (α-humulene) to 539.246 ± 133.002 [(Z) -3-hexenol acetate] (Figure 1). Mixture 2 elicited a stronger response than mixture 1 in both genders (F = 4.828, 1 df, P < 0.05).

There were great differences in response between the 28 compounds and two mixtures (F = 3.796, 27 df, P < 0.01), and,sex (F = 14.760, 1 df, P < 0.01). Among the GLVs, there were striking differences in the EAG responses to individual compounds between males and females. In males, the order of response was: 1-penten-3-ol < 2-penten-1-ol < (E) -2-pentenal < n-pentanol < (Z) -3-hexen1-ol < hexanol < (E) -2-hexenal < (Z) -3hexenol acetate. The response was significantly lower in females, the rank order of response to compounds being: 2-penten-1-ol < 1-penten-3-ol < (Z) -3-hexenol acetate < (E) -2-hexenal < (E) -2-pentenal < (Z) -3hexen-1-ol < hexanol < n-pentanol. Within this group, 1-penten-3-ol and 2-penten-1-ol elicited lower responses in both males and females. Within the group of aromatic compounds, acetophenone elicited significantly greater responses in males, while methyl salicylate elicited stronger responses in females. It was interesting to find that acetophenone and phenethyl alcohol elicited significantly stronger responses in this group. Among compounds belonging to the sesquiterpenoids, females were more sensitive to (+) -cedrol than males, but there were no distinct differences in response between (+) cedrol and α-humulen. Within monoterpenoids, the order of response in females was: geraniol < α-terpinene < nerol < (+) -3-carene < ocimene < a-terpineol linalool. In males, the order of response was: ocimene < (+) -3-carene < a-terpinene < aterpineol < nerol < geraniol < linalool. Multiple comparisons of relative EAG responses showed strong differences in response between compounds (F = 3.575, 25 df, P < 0.01) in males, but no significant differences in females (F = 1.468, 25 df, P > 0.05) (Figure 1).

#### Differentiation of EAG responses within different compound types

**Figure 2.**
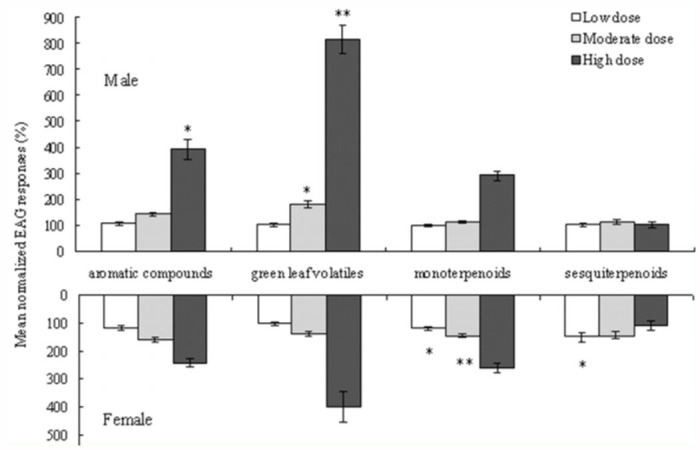
Mean normalized EAG responses of adult females and males of *lragoides fasciata* to different compound types, viz. aromatic compounds, green leaf volatiles, monoterpenoids and sesquiterpenoids. Responses are relative and expressed as a percentage of response to the standard linalool (mean±.E., n= 15). An asterisk indicates chemicals which elicited different relative EAG responses between males and females (^**^: p < 0.01, ^*^: p < 0.05). High quality figures are available online.

To make comparisons between groups of different compound types, EAG responses to individual compounds belonging to a particular compound type were collected and averaged. In general, there were great differences in response between compound types (F = 3.884, 3 df, P < 0.01). The males responded more strongly to GLVs (10^-4^ g/mL, F = 6.174, 1 df, P < 0.05 10^-2^ g/mL, F = 28. 980, 1 df, P < 0.01 ) and aromatic compounds (10^-2^ g/mL, F = 12.607, 1 df, P < 0.01 ) than females, while sesquiterpenoids (10^-6^g/ml, F = 14.832, 1 df, P < 0.01, 10^-4^ g/mL, F = 26.405, 1 df, P < 0.01) elicited stronger responses in females than in males (Figure 2).

#### Differentiation between dose responses of both genders

**Figure 3.**
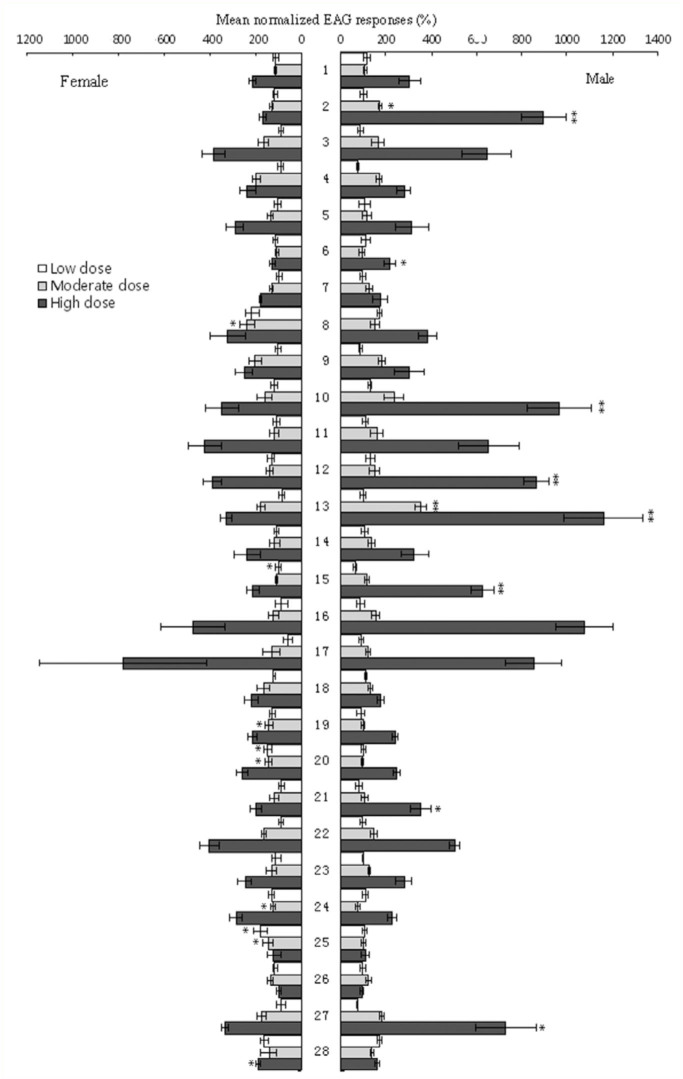
Mean normalized EAG dose-response property for female and male *lragoides fasciata* to volatile counpounds. Responses are relative and expressed as a percentage of response to the standard linalool (mean±S.E., n=5). An asterisk indicates chemicals which elicited different relative EAG responses between males and females (^**^: p < 0.01, ^*^: p < 0.05). High quality figures are available online.

Most tested compounds elicited dosedependent responses in both genders of *I. fasciata*. Analysis of dose responses between males and females of *I. fasciata* adults by paired t-tests indicated that there were no significant differences between genders (p > 0.05) for most compounds. The males responded more strongly to 2-penten-1-o1 (10^-2^ g/mL, P < 0.01), acetophenone (10^-4^ g/mL P < 0.05 10^-2^ g/mL, P < 0.01 ), (E) -2-hexenal (10^-2^ g/mL, P < 0.01), hexanol (10^-2^ g/mL, P < 0.05), indole (10^-2^ g/mL, P < 0.01), mixture 1 (10^-2^ g/mL, P < 0.05), (Z) -3-hexen-1-ol (10^-2^ g/mL, P < 0.01), (Z) -3-hexenol acetate (10^-4^ and 10^-2^ g/mL, P < 0.01), while the responses were weaker to (+) -cedrol (10^-6^ and 10^-4^ g/mL, P < 0.05), a-terpinene (10^-4^g/ml, P < 0.05), methyl salicylate (10^-4^g/ml, P < 0.05), mixture 2 (10^-2^ g/mL, P < 0.05), ocimene (10^-4^ g/mL, P < 0.05) (Figure 3).

### Discussion

Plants can emit a variety of volatile compounds, with molecular weights from 100 to about 200 Da, into the atmosphere around them (Schoonhoven et al. 2005). At present, over 1,000 low molecular weight organic compounds have been identified to be emitted from plants (Dudareva et al. 2004). With the olfactory receptor systems, phytophagous insects can perceive some of these plant volatiles, and exploit them as a chemical cue to find a suitable food plant, or a habitat for mating or ovipositing (Visser 1986; Dicke et al. 2000). Phytophagous insects show specialized feeding habits. In general, each species often limits its feeding to a limited range of taxonomically related plant species, or even to particular plant parts (Visser 1986). Two contrasting hypotheses have been proposed in regard to plant odor specificity: (1) Plant odors are highly specific, and composed of compounds not found in unrelated plant species, or (2) plant odor specificity is achieved by the particular ratio between constituent compounds, which are generally distributed among plant species (Visser 1986; Bruce et al. 2005). Overwhelming evidence has been discovered to support the latter. This study showed that both males and females of *I. fasciata* responded to the 26 plant volatiles. It is apparent that no single compound acts as a principal compound for host-plant selection in *I. fasciata*.

GLVs are volatile chemicals that green plants release. The proportion and composition of the components varies between different plant species (Visser et al. 1979). Phytophagous insects use the particular green odor blend of their host-plants to locate suitable feeding and oviposition sites. A specific blend of green leaf components comprising (E) -2-hexenal, (Z) -3-hexenyl acetate, (Z) -3-hexen-1-o1, and (E) -2-hexen-1-o1, and representing the odor of potato leaves, acts as an orientation cue for the Colorado potato beetle, *Leptinotarsa decemlineata* (Alexander et al. 1978; Visser et al. 1979; Thiery et al. 1986). In our study, substantial EAG responses were easy to find in the group of green leaf volatiles. This sensitivity suggested a species-specific adaptation of the set of olfactory receptors on the antennae to the particular green odor components of tea plant leaves. Thus, the GLV composition may be an important clue in host-plant selection by this oligophagous species. The stronger EAG responses in males of *I. fasciata* as observed in this study may occur through additive or synergistic effects.

The ability of both genders to detect plant volatiles is probably due to their similar habitat, which requires the use of the same clues to locate host-plants for survival and reproduction. The greater response in females, in comparison with males, to plant volatiles have been reported in many insects (Pers 1981; Ramanchandran et al. 1990; Raguso et al. 1996; Zhang et al. 1999; Das et al. 2007). It is possible that there are more olfactory receptor cells that are sensitive to plant volatiles in the female antennae (Raguso et al. 1996). In this study, greater response in females in low or moderate doses, elicited by some plant volatiles from aromatic compounds, sesquiterpenoids, and monoterpenoids, showed that these plant volatiles might be important olfactory cues in the host-plant location in the female moths. The greater response of males to GLVs has also been reported in some phytophagous insects (Raguso et al. 1998; Raguso et al. 1996). It is interesting to find that the male responded more strongly to most of the tested compounds than the female in moderate or high doses, while there were no significant differences between genders for most compounds in low doses. The antennae morphology observation of the moth under stereomicroscope found that the males had pectinate antennae, while the female had filiformis antennae (Huang et al. 2009). This indicates that the antennae in males have a greater surface area which allows easier detection of the semiochemicals. It is possible that there are more olfactory receptor cells that are sensitive to plant volatiles in the male antennae. More work is necessary, such as scanning electron microscopy of antennae of adult male and female *I. fasciata*, and identification of active plant volatiles using single-sensillum recordings from olfactory receptor neurons, to support this hypothesis.

## **Abbreviations**

EAG,: electroantennogram;
GLVs,: Green leaf volatiles;
IPM,: Integrated Pest Management
